# Female Doctors

**Published:** 1854-11

**Authors:** 


*London Lancet.*

---

*Female Doctors.*—All of us remember how profoundly, a few years ago, the female mind in this country was agitated by the Bloomer crusade. The cry went forth from the women of America to the women of England, calling upon them, as they valued their freedom —or, rather, their supremacy—to rebel against the yoke which was supposed to be typified in their petticoats; to revolutionize the time-honored but despotic millinery-regulations; and to put on, as the symbol of their independence and of their equality with tyrant man, that garment of the nether limbs which it is considered indelicate to describe by name, but which hitherto had been wrongfully and despotically appropriated to the male sex. The philosophical and skilful Amazon, Mrs. COLONEL BLOOMER, who had assumed the conduct of this great campaign on behalf of the rights of women, had accurately fathomed the influence of dress upon the mind. The ingenious author of "Sartor Resartus" himself did not more fully appreciate the importance of external decorations as instruments for subjugating the intellect of mankind, than did the gallant COLONELESS when she boldly unfurled before her down-trodden sisters, as the standard of revolt, that unmentionable garment which insolent men had oftentimes in ribald metaphor taunted them with surreptitiously wearing.

It is not our purpose to trace the succession of events by which the failure of the Bloomer costume was brought about. We still avow ourselves of the school of THOMAS CARLYLE. We still believe in the efficacy of dress as an agent in moral government. If the —— (our readers must supply the word we are not at liberty to mention) are still worn only by a few of the more daring and determined of their sex, we can only conclude that the petticoat which Mrs. BLOOMER would discard and repudiate as unfitted to be the emblem of government is, in reality, the garment that is the most in harmony with the mental qualities that Nature has implanted in the female sex. But contemporaneously with this bold assumption of masculine attire, there was at work, in the more advanced and disenthralled departments of the United States, a spirit of ambition more practical in its nature, that aimed at grasping not only the outward garments of man, but also at usurping functions which the tyranny of custom had assigned to him. Female Colleges of all kinds were instituted. The inherent right of women to academical distinctions —to enter upon the practice of the learned professions—was loudly proclaimed. Medicine, ever peculiarly exposed to the inroads of charlatanry, was the first profession to tempt the zeal and the ambition of the New Amazons. It had long been a subject of reproach that Medicine was degraded by the ignorant pretentions of "old women" of both sexes; why should it not be regenerated and honored by *young* women of powerful and instructed minds? Accordingly we have witnessed the revival of lady-professors of anatomy, physiology, medicine, surgery and midwifery; we have seen these lady-professors conferring the degree of DOCTOR of Medicine—we believe they pretermit the lower grade of BACHELOR—upon the young ladies who have completed their studies; and, lastly, we have seen these lady-dubs, under their proper professional and academical appellations, driving about the enlightened towns of America in befitting broughams, expounding medical mysteries, giving advice and taking fees! One of these lady-doctors, more ambitious than her professional sisters, not satisfied with the state of science in America, has laudably resolved upon improving her learning and her skill, by visiting the European Schools of Medicine.

A Miss Doctor BLACKWELL, a Graduate of Cleveland College, Ohio, having completed a course of clinical study at the Royal Maternity Hospital, at Edinburgh, has applied to the Managers of the Royal Infirmary for permission to prosecute her studies in the female wards of that institution. It is supposed that her intention is ultimately to eclipse the home-bred lady-graduates of America, by returning to New York with the degree of Doctor of Medicine of the University of Edinburgh, and the Fellowship of the Royal College of Physicians of London. That a purpose so praiseworthy should be opposed by the refusal of the Managers of the Royal Infirmary to accede to the request of Miss Doctor BLACKWELL, we cannot but regret. In order that Miss Doctor BLACKWELL may not return to America, there to propagate the injurious reproach against our national character, that we know not how to encourage females in the pursuit of medical knowledge and collegiate honors, we beg leave respectfully to inform her and her sister-graduates, that the Royal College of Surgeons of England is animated by a more enlightened and transatlantic liberality; and that the new order of Licentiates in Midwifery is open, by an Act of Parliament, to "persons" of both sexes.—*London Lancet.*

